# Prevalence of HIV Drug Resistance Mutations in HIV Type 1 Isolates in Antiretroviral Therapy Naïve Population from Northern India

**DOI:** 10.1155/2012/905823

**Published:** 2012-03-15

**Authors:** S. Sinha, H. Ahmad, R. C. Shekhar, N. Kumar, L. Dar, J. C. Samantaray, S. K. Sharma, A. Bhargava, R. M. Pandey, R. L. Mitsuyasu, J. L. Fahey

**Affiliations:** ^1^Department of Medicine, All India Institute of Medical Sciences, Ansari Nagar, New Delhi 110029, India; ^2^Department of Microbiology, All India Institute of Medical Sciences, Ansari Nagar, New Delhi 110029, India; ^3^Department of Biostatistics, All India Institute of Medical Sciences, Ansari Nagar, New Delhi 110029, India; ^4^UCLA Center for Clinical AIDS Research and Education, University of California, Los Angeles, CA 90035, USA; ^5^Department of Medicine and Microbiology, University of California, Los Angeles, CA 90095, USA

## Abstract

*Objective*. The increased use of antiretroviral therapy (ART) has reduced the morbidity and mortality associated with HIV, adversely leading to the emergence of HIV drug resistance (HIVDR). In this study we aim to evaluate the prevalence of HIVDR mutations in ART-naive HIV-1 infected patients from northern India. *Design*. Analysis was performed using Viroseq genotyping system based on sequencing of entire protease and two-thirds of the Reverse Transcriptase (RT) region of *pol* gene. *Results*. Seventy three chronic HIV-1 infected ART naïve patients eligible for first line ART were enrolled from April 2006 to August 2008. In 68 patients DNA was successfully amplified and sequencing was done. 97% of HIV-1 strains belonged to subtype C, and one each to subtype A1 and subtype B. The overall prevalence of primary DRMs was 2.9% [2/68, 95% confidence interval (CI), 0.3%–10.2%]. One patient had a major RT mutation M184V, known to confer resistance to lamivudine, and another had a major protease inhibitor (PI) mutation D30N that imparts resistance to nelfinavir. *Conclusion*. Our study shows that primary HIVDR mutations have a prevalence of 2.9% among ART-naive chronic HIV-1 infected individuals.

## 1. Introduction

The national antiretroviral therapy (ART) program in India for treatment of human immunodeficiency virus (HIV) and acquired immune deficiency syndrome (AIDS) was started by National AIDS Control Organization (NACO), Ministry of Health and Family Welfare, Government of India, in April 2004. By the end of November 2009, more than 2, 50,000 patients infected with HIV had received ART under the program [1]. As per the latest report by Joint United Nations programe on HIV/AIDS (UNAIDS), prevalence of HIV in India is estimated to be 0.31%, that translates to approximately 2.31 million persons living with HIV/AIDS [2]. The current standard first-line treatment for HIV in India consists of two nucleoside reverse transcriptase inhibitors (NRTIs), zidovudine or stavudine plus lamivudine, and one nonnucleoside reverse transcriptase inhibitor (NNRTI), nevirapine or efavirenz. Regimens with protease inhibitors (PIs) are available as second-line treatment options upon failure of the first-line ART under the national program. As per the time trends for evolution of primary HIVDR suggested by Grant et al*., *it can be expected that the prevalence of drug resistance mutations (DRMs) may soon increase in India [3, 4]. The widespread use of ART has resulted in an increased prevalence of drug-resistant HIV strains, ranging from 10% to 20% among drug-naive patients in other countries [5–7]. The emergence HIVDR is inevitable, given the high replication and mutation rates of HIV, and the necessity for lifelong ART, which may not always be available to HIV-infected individuals in resource-limited settings, due to multitude of practical and logistic barriers. The genetic diversity of HIV-1 originates from rapid viral replication in infected individuals with a high rate of incorrect nucleotide substitutions in the viral genome. DRMs result from selective pressure during viral replication, especially in the presence of subtherapeutic levels of ART [8, 9].

Due to the predominance of subtype C in India, and limited data on nonsubtype B circulating HIV strains, studies to assess baseline, pretreatment DRMs in individuals infected with HIV-1 clade C are warranted [10]. It has also been established that patients who started their first-line ART regimen determined by baseline HIV genotypic testing have greater and longer lasting HIV viral suppression with therapy than those without HIVDR genotyping [11, 12]. Given the high cost and handling expertise for the genotyping assay in a resource-limited setting, prevalence studies are helpful in providing trends about HIV subtypes and the pattern of DRMs from a region. A recent study done at Mumbai in Western India by Deshpande et al. [13] included about 50 ART naïve HIV patients and showed 9.6% prevalence of DRMs. Similarly, another study done by Thorat et al. [14], from Kakinada in southern India, has shown the prevalence of DRMs to be <5% in HIV-infected ART-naïve population.

The present study discusses the prevalence of DRMs existing in treatment-naïve HIV-1-infected individuals from northern India and assesses the subtypes prevalent in these individuals.

## 2. Material and Methods

### 2.1. Patient Population

All HIV-infected patients visiting Integrated Counseling and Testing centre (ICTC) and ART clinic at All India Institute of Medical Sciences (AIIMS), New Delhi, were screened for eligibility. They had been confirmed for HIV-1 seropositivity by three sets of Enzyme-linked Immunosorbent Assay (ELISA) according to NACO guidelines [15, 16]. Detailed treatment history was taken from all patients. Those who reported no prior exposure to antiretroviral drugs were considered ART naïve. Only adult (>18 years of age) patients with CD4 cell counts below 200/*μ*L were considered for entry into the study. The study was approved by AIIMS ethics committee and written informed consent was taken from all study participants. Seventy-three chronic HIV-1-infected ART-naïve individuals, who were eligible for first-line ART, were enrolled from April 2006 to August 2008. DNA was successfully amplified and sequenced in plasma samples from 68 patients.

### 2.2. Specimens

Ten milliliters (mL) of whole blood sample were taken from each patient. Three mL were used for CD4+ T cells count estimation, and the remaining was centrifuged within 6 hours of collection at 400 g for 10 minutes in order to separate plasma. Plasma was distributed into 1 mL aliquots and stored frozen at −70°C. Fresh aliquots of plasma were used for HIV-1 RNA quantification and HIV-1 genotyping as per the WHO and HIVResNet Laboratory Working Group resistance testing guidelines [17].

### 2.3. Viral Load Testing and CD4 Estimation

Viral load testing was performed using the standard protocol of AMPLICOR HIV-1 Monitor Test, version 1.5 (Roche Molecular Systems Inc., Branchburg, NJ, USA). CD4/CD8+ T cell counts were determined by flow cytometry using BD FACS CALIBUR (BD Biosciences, CA, USA).

### 2.4. HIV-1 Genotyping

HIV-1 genotyping was performed using the Abbott ViroSeq HIV-1 Genotyping Systems (Abbott diagnostics, Wiesbaden, Germany) to sequence the 1.8 kb protease-RT region of HIV-1 *pol *gene as per standard procedure [17]. RNA extraction was performed on 500 microlitre (*μ*L) of plasma using the guanidine-thiocynate extraction method. A reverse transcription polymerase chain reaction (RT-PCR) followed by PCR was carried out to generate an amplicon of 1.3 kb. The amplicons were purified using silica spin columns, and PCR products were run on 1% agarose gel against 2 mass ladders allowing for semiquantitation of DNA. For sequencing, DNA was diluted according the band intensity on agarose gel, and PCR product bands with DNA >20 nanogram were selected. The latter were added to a 96-well reaction plate containing premixed Big Dye sequencing primers A, B, C, F, G, and H [17]. Sequencing was indigenously carried out in the Department of Microbiology, AIIMS, on the 16 capillary automated ABI PRISM 3130*xl* Genetic Analyzer (Applied Biosystems, Foster City, CA, USA) using data collection software v3.0 and sequence analysis software v5.3. ViroSeq HIV-1 Genotyping System Software v2.8 was used to assemble the chromatographs from the seven primers into a single project, and to generate a contiguous sequence spanning the entire protease gene, and up to codon 335 of the reverse transcriptase (RT) gene. This consensus sequence was compared to a known reference strain, HXB-2, to identify points of variance. The sequences were manually edited and saved in FASTA format which was submitted to Stanford HIV RT and Protease sequence database [18] to determine the drug-resistance profile and subtype of each sample. DRMs were defined according to WHO Surveillance mutation list 2009 proposed by Bennett et al. [19].

### 2.5. Quality Control

For quality control of HIV-1 genotyping, negative, low-positive, and high-positive control samples were run with every batch. The positive controls ensured the RT-PCR and genotyping success. To ensure good sequence quality, the high-positive control was sequenced before genotyping the HIV-1 clinical samples, precluding editing mistakes. Phylogenetic analysis was performed to check for contamination as per procedures described in the Los Alamos website (http://www.hiv.lanl.gov/) [20].

### 2.6. Clade Typing and Phylogenetic Tree

HIV-1 subtype was defined using REGA HIV-1 subtyping tool from Stanford HIV drug-resistance database (http://hivdb.stanford.edu/) [18], Worldwide subtype references were obtained from the Los Alamos HIV database [20]. For phylogenetic study, nucleotide sequences were aligned using software's GeneDoc 8 and Clustal X version 1.83 multiple sequence alignment programe. Genetic distances at nucleotide and amino acid level were calculated using MEGA software v4.0. The neighbor-joining method and kimura two-parameter model were used for tree construction with reliability estimated from 1000 bootstrap replicates [21, 22].

### 2.7. Statistical Analysis

Data was first recorded on a predesigned paper form and subsequently transferred to Microsoft Excel spreadsheet. All the entries were checked for possible keyboard error(s) at the entry level. The electronic data was exported into the STATA software v11, for statistical analysis. Baseline clinical and biological characters of the study subjects were summarized as frequency (%) for the categorical variables; and mean ± standard deviation (SD) or median {Interquartile  range  (IQR)} for quantitative variables. Prevalence of DRMs was computed with 95% confidence interval (CI).

## 3. Results

**Figure 1:**
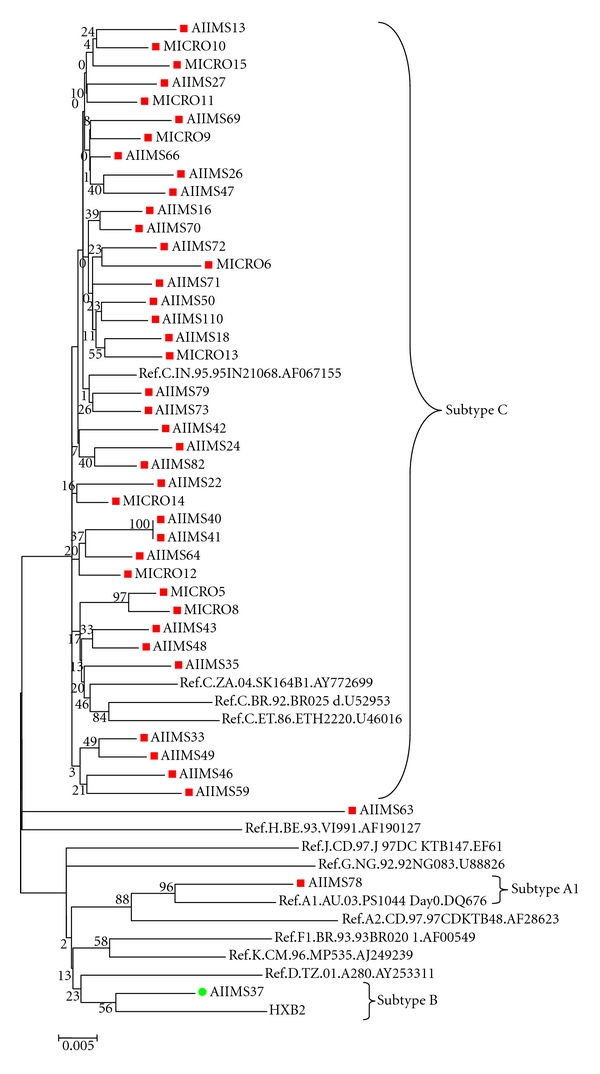
Phylogenetic relationship based on *pol* gene (1302 nt) sequences (shown in red and green color) and global HIV-1 subtype reference sequences (A1, A2, HXB2, C India, C Zambia, C Ethiopia, C Brazil, D, F, G, H J, and K from Los Alamos HIV Sequence Database).

A total of 73 patients were recruited into the study. Genotyping was possible in plasma samples from 68 (93%) of the participants. Five samples failed PCR amplification, of which 3 had viral load below 1000 copies/mL, and for the remaining two, reasons were unknown. Of the study participants, 91.2% gave a history of heterosexual exposure, 4.4% had bisexual behavior, and the rest did not reveal their HIV exposure history. Subtype C was found to be the most predominant subtype (97%) in our population (Table 1 and Figure 1). The median age of the study group was 35 years (range: 20–55 years). The median CD4 count was 107 cells/*μ*L (range: 49–157cells/*μ*L), and the median plasma HIV RNA load was 223640 copies/mL (5.34 log_10_), range: 8264 (4.98 log_10_)–750000 (5.75 log_10_).

Major HIV drug-resistance mutations were isolated from two of 68 patients (2.9%; 95% CI, 0.3%–10.2%). One patient (1.47%) had RT mutation, M184V that imparts resistance to NRTIs, lamivudine, and emtricitabine, and another (1.47%) had a major PI mutation, D30N, conferring resistance to nelfinavir. No NNRTI mutations were observed in our study (Table 2). Accessory minor PI mutations K20R, M36I, and H69K were seen in 7.3% (5/68), 97% (66/68), and 49% (33/68) patients, respectively; L63P, A71E, A71V, I13V, L10V, K45I, and K45R were observed in one patient each. 

## 4. Discussion

This is the first study sponsored by NACO for estimation of HIVDR mutation in ART-naïve population from northern India. Its results reveal overall prevalence of primary HIVDR to be 2.9% (CI, 0.3%–10.2%) in this region. As per WHO guidelines, the drug-resistance prevalence in a geographical area has been categorized into <5%, 5–15% and >15% [23]. This classification signifies the level of HIVDR surveillance programs required for monitoring primary HIVDR. An earlier study done by Chaturbhuj et al. [24] in 2010 has shown that the presence of surveillance DRMs in ART-naïve HIV-1-infected individuals recruited from Voluntary Centre for Testing and Counselling (VCTC) was less than the WHO threshold of 5%. Our data fits in the WHO low zone of (<5%), suggesting that primary HIV drug resistance is still under the limits in northern India, nevertheless there is a need for more data on primary drug resistance in ART naïve individuals.

In our study M184V, an RT mutation, which confers resistance to lamivudine was isolated in one case. Though this mutation is found most commonly in patients failing ART, it is rarely seen in ART-naïve patients as a transmitted drug-resistance (TDR) mutation [25]. This may be due to the diminished replicative capacity of the virus carrying this mutation, which increases the probability of the virus to get reverted back to the wild type. In earlier studies from India, M184V was found in two of 128 (1.6%) cases by Deshpande et al. in 2004 [26], and in one of 40 (2.5%) cases by Lall et al. 2009 [27]. No NNRTI mutations were detected in our study, which is consistent with the findings of Deshpande et al. [26].

One participant (1.5%) in our study had a major PI mutation, D30N, in the protease gene. This finding is close to that of Hira et al., who have shown a prevalence of 2.5% for major PI DRMs in ART-naïve HIV-infected population in India [28]. However, prevalence of the same has been reported to be as high as 20% in south India by Balakrishnan et al. [29], and 14.2% by Arora et al. [30] in north India. Since PI-based regimens are used less frequently in this country and have been distributed under the national program only after 2008, the prevalence of major PI mutation in the population may be assumed to be below 5%, as also corroborated by Chaturbhuj et al. in 2010 [24]. The possible reasons for reporting the higher DRMs by a few of the earlier studies may be the confusion over proper definitions of primary and TDR mutations, and nonuniformity between protocols for HIVDR surveillance until WHO criteria were published in 2009 [19].

One of the limitations of our study was that the study was designed and carried out at a time when WHO guidelines were not available and patients were tested at the pretreatment baseline rather than at the time of diagnosis. Hence, some of the mutated HIV strains may have reverted to the wild type, and we may have underestimated the prevalence of primary HIVDR [31, 32]. The other reason for underestimation of mutations could be that the sensitivity of detection of minority species by standard genotypic methods using population-based sequencing is only about 20% [33]. Another limitation of study is the small sample size, as HIV genotyping is costly, and limited funding was available for the study. A third limitation is that all patients may not have been ART-naïve, as chronically infected individuals often do not want to admit to previous treatment [34].

Our observation shows that 97% of the patients were infected with subtype C of HIV type 1. The subtype C strains clustered together along with the Indian reference strain (95IN216068). Previous studies performed in different parts of the country also corroborate the predominance of HIV-1 subtype C strains in India [35, 36]. Interestingly, along with subtype C, one stnain each of subtype B and subtype A1 were also found in our patient population. Similar findings have been reported earlier by Baskar et al. [37], and Gadkari et al., respectively [38].

In conclusion, our study reveals that the prevalence of primary HIV DRMs is 2.9% in northern India, which is within the WHO threshold limit of <5%. This finding reinforces the national program's effort in maintaining low level of primary drug resistance among HIV-infected population. Due to the limited options for second- and third-line ART in a resource-constrained setting, baseline HIV genotyping is required for better patient monitoring and keeping HIVDR in check. However, until the procedure becomes more affordable, there is a need for continued surveillance and further prevalence studies with larger sample sizes to assess primary drug resistance patterns in the Indian HIV-infected population. 

## Figures and Tables

**Table 1:** Baseline clinical and biological characteristics of the 68 study subjects.

Variables	Summary, *n* = 68
Age(yrs), median (IQR)	35 (30–40)
Gender, *n* (%):	
Male	61 (89.7%)
Female	07 (10.3%)
Median CD4Tcell count, cells/*μ*L (IQR)	107 (49–157)
Median Viral load, log_10_ copies/mL (IQR)	5.34 (4.98–5.75)

Viral load (log_10_ copies/mL), *n* (%):	
<4.0	2 (2.9%)
4.0–4.9	14 (20.6%)

≥5.0	52 (76.5%)

Risk exposure, *n* (%):	
Heterosexual (%)	62 (91.2%)
Bisexual (%)	3 (4.4%)
Others/unknown (%)	3 (4.4%)

Other coinfections, *n* (%)	57 (83.8%)

HIV-1 subtypes, (%):	
Subtype C	97%
Subtype A1	1.5%
Subtype B1	1.5%

Values are expressed as number (%) or median (Interquartile range).

**Table 2:** Data of two study participants detected with NRTI and PI drug-resistance mutations.

Study ID	CD4 count (cells/*μ*L)	Viral load (copies/mL)	HIV-1 subtype	Drug resistance mutations by antiretroviral class*	
				NRTI	PI
AIIMS 10	157	63721	C	M184V	—
AIIMS 81	77	>750000	C	—	D30N

*Standard single-letter amino acid codes indicate mutations. NRTI: nucleoside reverse transcriptase inhibitor; PI: protease inhibitor.
